# Enzyme-assisted extraction of antioxidative phenolics from grape (*Vitis vinifera* L.) residues

**DOI:** 10.1007/s13205-012-0055-7

**Published:** 2012-04-08

**Authors:** Ricardo Gómez-García, Guillermo C. G. Martínez-Ávila, Cristóbal N. Aguilar

**Affiliations:** 1 Unidad Saltillo, Departamento de Investigación en Alimentos, Facultad de Ciencias Químicas, Universidad Autónoma de Coahuila, 25280 Coahuila, Mexico; 2 Laboratorio de Biotecnología, Facultad de Agronomía, Universidad Autónoma de Nuevo León, 66050 Escobedo, NL Mexico

**Keywords:** Grape waste, Enzyme technology, Polyphenolic compounds

## Abstract

Agro-industrial byproducts represent a serious environmental problem and the industries producing these residual materials have incurred expenses for their proper disposal and generally increase the pollution due to the high content of organic substances and might represent legal problems. However, the residues such as grape wastes are potential source of phenolic compounds which are widely known for their high antioxidant activity. Bioprocesses such as enzyme technology represent an alternative for production of those bioactive compounds from agro-industrial byproducts. In this study, different types of commercial enzymes such as Celluclast^®^ 1.5 L, Pectinex^®^ Ultra and Novoferm^®^ were used to release phenolic compounds from grape wastes. The hydrolysates were analyzed in their total phenolic compounds and antioxidant activity with Folin–Ciocaletu test and DPPH· radical-scavenging assay, respectively. A good correlation was obtained between antioxidant activity and phenolics released. The highest antioxidant activities registered were 86.8 ± 0.81, 82.9 ± 0.31 and 90 ± 0.37 % at 12 h for Celluclast^®^ 1.5 L, Pectinex^®^ Ultra and Novoferm^®^, respectively. Novoferm^®^ had the strongest effect on phenolic release from grape waste, followed by Pectinex^®^ Ultra and Celluclast^®^ 1.5 L. High performance liquid chromatography–electrospray–mass spectrometry clearly revealed that the increment of antioxidant activity is associated with the release of *O*-coumaric acid.

## Introduction

Phenolic compounds are the most abundant antioxidants in the fruits and plant-derived beverages such as fruit juices, tea, coffee, and red wine. Health effects of dietary polyphenols have come to the attention of nutritionists only rather recently. Citrus peels, pomegranate husk, raspberry pomace and grape waste are a rich source of phenolic compounds, which are widely known for their high antioxidant and free radical-scavenging capacity (Baydar et al. 2007; Jayaprakasha et al. 2003; Starzynska-Janiszewska et al. 2008; Vattem and Shetty 2003).

Grape is the most widely cultivated fruit crop in the world, with a global production of around 69 million tons. About 80 % of the total amount is used in wine-making (Maier et al. 2009), generating huge quantities of grape wastes, which considerably increase the pollution due to high content of organic substances, resulting in damage of biological life in discharged zones.

Some chemical processes have been employed to obtain polyphenolic compounds from different residual sources including grape wastes (Sultana et al. 2008; Negro et al. 2003). However, due to the unspecificity of these reactions, chemical procedures have large problems for the recovery of those compounds, thus generating low yields; also they are of high environmental impact and in some cases they represent a hazard to human health. In recent years, biotechnological hydrolysis for bioactive compounds production has attracted more attention. Enzyme-assisted extraction represents a potential bioprocess for the production/extraction of phenolic compounds with high stability and high antioxidant activity for pharmaceutical and food applications. Moreover, enzyme-assisted extraction permits the utilization of agro-industrial byproducts providing a novel alternative to give added-value to those non-utilized residues.

According to Li et al. (2005), the mechanism for enzyme-assisted extraction of phenolic compounds from residual sources is based on the cell-wall degrading enzymes that can weaken or break down the cell wall, leaving the intracellular materials more exposed for extraction. Nonetheless, the presence of polyphenolic compounds associated with cell-wall polysaccharides in grapes have been reported (Pinelo et al. 2006). In this way, the break down or partial degradation of cell-wall polysaccharides also permits the extraction of those bioactive compounds (Li et al. 2005; Robledo et al. 2008; Bhanja et al. 2008).

This study was carried out to determine the potential power of the hydrolysates of grape waste obtained by enzyme-assisted extraction for scavenging the stable DPPH· free radical.

## Methodology

DPPH·, gallic acid and acetonitrile (HPLC grade) were purchased from Sigma-Aldrich. Others chemicals were analytical grade.

### Raw material and enzyme-assisted extraction of phenolics

Grape solid waste was obtained from a wine-making industry in the state of Coahuila (North of Mexico). The material was dehydrated at 60 °C for 24 h and then pulverized to a 30 mesh particle size. Grape waste was stored at room temperature protected from sun light until its analysis.

Enzymatic hydrolysis was carried out as follows; 100 mg of dry material was suspended in 1.4 mL of 0.2 M acetates buffer (pH 3.5). Later on, 100 μL of a dilution (1:10) of the respective enzymatic preparations was added to the mixture. Afterward, the samples were incubated in a Termomixer (Labnet S2056-A) at 40 °C, and then the treatments were monitored at 0, 1, 3, 12, 24, 36 and 48 h. To stop the enzymatic hydrolysis, the hydrolysates were placed in a water bath at 100 °C for 5 min and the hydrolysates were filtered through a Whatman No. 41 filter paper. Obtained extracts were analyzed for their phenolic content and scavenging capacity. All the determinations were made by triplicate and the means were reported.

### Folin–Ciocalteu assay and DPPH· free radical-scavenging assay

Polyphenolics content was determined by Follin–Ciocalteu assay (Makkar et al. 1993). DPPH· (1,1-diphenyl-2-picrylhydrazyl) radical scavenging was carried out according to the methodology proposed by Randhir and Shetty (2007). The radical-scavenging capacity of the extracts was calculated with the following equation and expressed as DPPH· percent of inhibition:HPLC–ESI–MS analysis of samples before and after the enzymatic treatment was performed to identify the phenolics released during the enzyme-assisted extraction from grape wastes. HPLC analysis was carried out in a Varian Pro-Star 330 system (Instrumentación Analítica S.A. de C.V., Monterrey, Nuevo León, México). A photodiode array (PDA) detector with detection of 280 nm was used. Fractionation of the injected material was carried out on an Optisil ODS column (5 μm, 250 × 4.6 mm) at 30 °C. A gradient mobile phase consisting of acetonitrile (solvent A) and acetic acid at 3 % v/v (solvent B) was applied at a flow rate of 1 mL/min. Sample injection volume was 10 μL. Then, samples were analyzed in a Varian 500/MS IT Mass Spectrometer, using electrospray ionization with a positive ionization was under a run of 7 min for polyphenolic compounds detection. Mass detection was in a range from 100 to 300 (*m*/*z*).

## Results

**Fig. 1:**
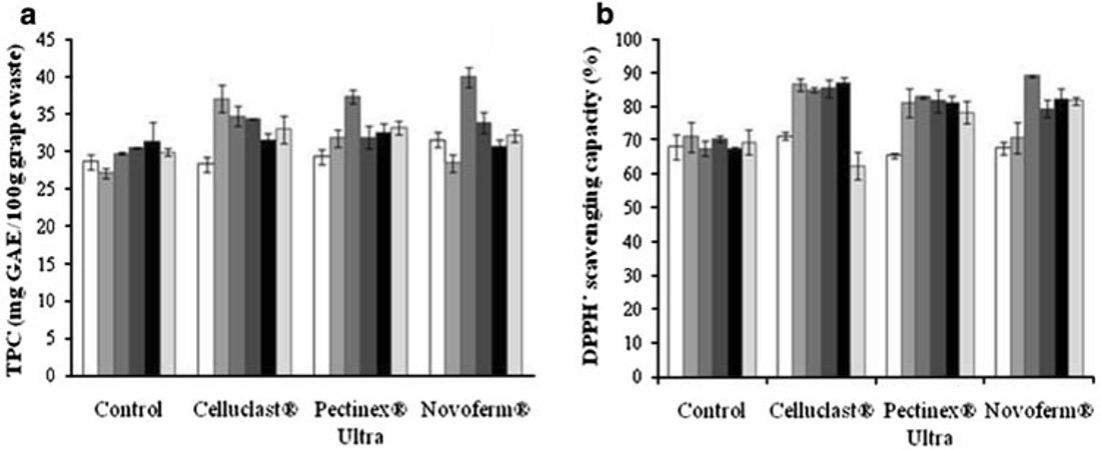
**a** Total phenolic content and **b** DPPH· scavenging capacity ( ) 0 h; ( ) 1 h; ( ) 12 h; ( ) 24 h; ( ) 36 h; ( ) 48 h. Results are presented as mean values ± SD for triplicate analyses

Grape waste is a potential source of polyphenolic compounds, which are well known for their multifunctional properties. Some of the main aspects about it cell-wall structure, chemical composition and parameters that affect the yield of polyphenolics, which must be considering for enzyme-assisted extraction, have been reported in the literature (Pinelo et al. 2006, 2008). Figure 1a, shows the effect of incubation time on the phenolic content in the hydrolysates of grape waste obtained using three different enzymatic commercial preparations. An incubation period of 12 h was sufficient for enzyme-assisted extraction of phenolic compounds. The highest releasing of phenolics present in the grape waste was obtained with Novoferm^®^ which released more than 25 % with respect to the control. Our findings are consistent with those previously reported by Meyer et al. (1998), whom observed an increment in phenolic compounds after enzymatic hydrolysis of grape pomace. The authors found that phenolics releasing depends on type of enzyme, time of enzymatic treatment, particle size of the pomace, and type of extraction solvent employed in their experimentation. On the other hand, not only grape waste has been studied for enzymatic polyphenolic releasing form residual sources. For example, Li et al. (2005) reported the highest amounts of phenolic compounds (approx. 140 mg/100 g) on grapefruit extracts after hydrolyzing with Cellzyme MX, which was 28 % higher than the control. In addition, it was reported that content of polyphenolics was increased in 35 % when the effect of enzymatic treatment temperature during the hydroethanolic extraction of phenolic antioxidants from raspberry residues was evaluated (Zuniga-Hansen and Laroze 2009). This confirms that the enzyme-assisted extraction is one of the more effective techniques to increase phenol yields since it improves the recovery of phenols from agro-industrial byproducts. We can consider that enzyme-assisted extraction is an attractive alternative for recovery of bioactive compounds from grape fruit.

Several chemical processes have been developed to evidence the high scavenging capacity of grape waste extracts (Jayaprakasha et al. 2003; Starzynska-Janiszewska et al. 2008; Yemis et al. 2008). Nonetheless, those procedures could be expensive and generate large amount of contaminants. For this study, all the hydrolysates of grape waste kinetically obtained from the enzyme-assisted extraction were tested for their scavenging capacity with DPPH· free radical. Figure 1b shows the capacity of extracts of grape waste for scavenging this synthetic stable free radical. In concordance with polyphenolics content, the hydrolysates from grape waste obtained after treatment with Novoferm^®^ showed the highest scavenging for the DPPH· radical at 12 h. A significant difference was not observed for scavenging such free radical with the hydrolysates obtained from 1 h to 36 h when Celluclast^®^ and Pectinex^®^ Ultra were tested. However, DPPH· radical scavenging ranged from 81 to 90 % which were higher than the control. In presence of antioxidant substances, free radical scavenging should increase until all the antioxidant compounds present in the samples are sacrificed. In this sense, we can assume that phenolic compounds in the analyzed samples are responsible for scavenging of the DPPH· free radical. Our findings were comparable with those extracts from grape waste obtained chemically using methanol and ethanol (Lafka et al. 2007) and acetone:water:acetic acid mixtures (Jayaprakasha et al. 2003). Moreover, the values for DPPH· radical scavenging recorded for the hydrolisates in this study were higher than those reported for raspberry pomace after its treatment at similar conditions (Zuniga-Hansen and Laroze 2009).

**Fig. 2:**
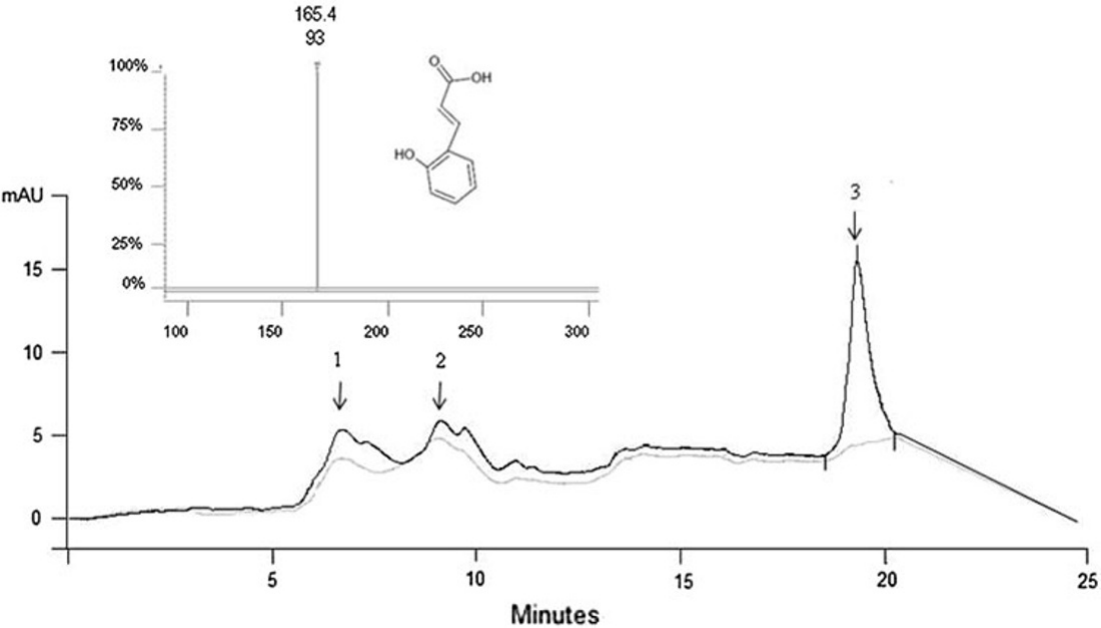
HPLC–ESI–MS profiles of grape waste before (*gray line*) and after (*black line*) the best enzymatic treatment (Novoferm^®^ 12 h). *1* gallic acid, *2* resorcinol, *3O*-coumaric acid

Figure 2 shows the HPLC profiles and the MS analysis of the treated and control samples. Although small signals corresponding to gallic acid and resorcinol were detected in the samples, HPLC–ESI–MS revealed that the increment of antioxidant activity was clearly associated with release of *O*-coumaric acid from the cell-wall fibers, where is located in the fruits. Our findings are consistent with those reported previously by Lafka et al. (2007), whom evidenced that *O*-coumaric acid is one of the most important phenolic compounds present on winery wastes. Although *O*-coumaric acid was the main phenolic compound recorder, it is important to mention that other phenolic compounds could be present in the samples. For that reason it is necessary for an identification of all the phenolics related with the free radical scavenging of the analyzed extracts.

## Conclusions

In last few years, biotechnology strategies to obtain antioxidant substances have been received special attention. In this work, the assisted-enzyme extraction as an alternative for extraction of phenolic antioxidants from winery wastes was evaluated. Thus, phenolic compounds can be considered to be added-value from industrial wastes, justifying their isolation from residual sources and this way to give utilization to this material.
